
JOURNAL INFORMATION
==============================
NLM Title Abbreviation: Med Chir Rev
NLM Journal ID: 0034617

The Medico-Chirurgical Review

Publisher: Biomedical Journal Digitization Project

ARTICLE INFORMATION
==============================
PMCID: PMC4966264
PMID: 29917634
Article version: 1
Subjects: Reviews

Critical and Analytical Review of Erysipelas

Print publication date: 1828 Oct 1
Volume: 9
Issue: 18
First page: 361
Last page: 384
1828 Aug
License: This work is free of known copyright restrictions. For more information, please see PMC Back Issue Digitization.
License URL: https://creativecommons.org/publicdomain/mark/1.0/

==============================

### X.

1. *Observations on the Nature and Treatment of Erysipelas, illustrated by Cases.* By W. Lawrence, Esq. F.R.S. &c.—(*Medico-Chirurgical Transactions, London, Vol.* XIV.)

2. *Account of the Epidemic Erysipelas which appeared at Montrose and the Neighbourhood in* 1822. By W. Gibson, M.D.—(*Medico-Chirurgical Transactions, Edinburgh, Vol.* III. *p.* 94.)

3. *On the Treatment of Erysipelas by Numerous Punctures.* By R. Dobson, M.D.—(*Medico-Chirurgical Transactions, Vol.* XIV.)

4. *Case of Erysipelas, with some Remarks.* By A. Copland Hutchison, Esq.—(*Medico-Chirurgical Transactions, Vol.* XIV.)

The atmosphere of medicine is as easily agitated as that of nature—the vicissitudes which it experiences are as diversified and as multifarious—and although the effects produced are not as momentous in their character, they are neither less curious nor less useful. The uninteresting sameness of routine practice is occasionally varied by the stormy discussions of rival spirits, and the discovery of some new, or the revival of some old improvement, imparts an impulse to our stagnating powers, fans the flame of genius into increased activity, and accelerates "the march of intellect." Such discussions prove of the utmost service to our profession; for, whether they end by augmenting or diminishing our store of remedies, they must issue in improvement.

During the late meetings of the Medico-Chirurgical Society, a very warm but interesting debate succeeded the reading of the Essay on the Nature and Treatment of Erysipelas, which now occupies our notice; and although personal invective occasionally intermingled with the argument, the importance of the subject excited much attention, and elicited many valuable observations.

The principal objects of this very learned and laboured essay are two;—to inculcate the identity of erysipelas and inflammation, and to recommend, in the treatment of phlegmonous erysipelas, the employment of incisions, as the most speedy and effective mode of cure.

It is well known that erysipelas was considered an atonic affection by some of the most experienced writers of antiquity, and that the doctrine of its essence being inflammation in all cases, is impugned in the works of many modern authors. Hippocrates believed it to arise from acrid humours; Galen, speaking of its diagnosis, says, "non tamen similiter dolet erysipelas ac inflammatio:" and, in obedience to the opinions of these medical fathers, almost every succeeding writer of distinction, until the appearance of La Motte, advocated its humoral pathology. Sennertus says, from genuine inflammation (a vera inflammatione) it likewise differs, because it is accompanied by less tumefaction, pulsates less, and is less tense and shining. But many diseases, which were held to be diseases of debility by the ancients, have since either changed their nature, or are treated in the present day as though they had; and Mr. Lawrence stands eminently forward in the van of those, who see strength in almost every malady, and thinks the lancet as essential an article of furniture in the sick chamber as the nurse or the night-stool.

Although, however, we hold ourselves quite exempt from the charge of timidity, and can bleed with fearlessness when symptoms demand, we attach no such unlimited importance to this instrument, because we subscribe not to that antiphlogistic creed, which teaches its heroic votaries to wear it as their crest.

"Regarding it," says Mr. Lawrence, "as an affection essentially inflammatory, some adopt the antiphlogistic plan, including general and local bleeding; while others, conceiving that the part, the constitution, or both, are in a state of debility, endeavour to remove this by the free use of stimulants and tonics, more especially by bark, ammonia, and wine. The former appears to me the correct view and practice; I accordingly consider the latter notion completely erroneous, and the treatment founded on it, not only inappropriate, but injurious." 2.

That erysipelas is, *in one sense* of the expression, always inflammatory, there can be no doubt, since many of its external characters are those which accompany and constitute inflammation; but the question and the difficulty are, whether these inflammatory characters are, *in every sense, purely phlogistic*, and invariably admit or require for their removal antiphlogistic measures. It will be admitted that inflammation may exist, and yet the lancet be improper. The pulse may be low, and the constitution may be weak; or the circulation, although excited by the local disease into simulated power, may not endure depletion. The co-existence of local inflammation and general debility is quite compatible with orthodox pathology, and is often realized in actual practice. How frequently do we witness erysipelas supervening upon the close of acute diseases, when the powers of life have been nearly exhausted by active treatment and protracted illness, and when the most strengthening remedies are required and often prove insufficient. In many instances, debility constitutes the essence of erysipelas, and in every case, we believe, there is so much admixture of symptom and of character, as entitles us to hold phlegmonous and erysipelatous inflammations to be essentially different. They attack different habits, invade different textures, are marked by different symptoms, proceed in different stages, admit of different treatment, and issue in different terminations. When we admit that they are bound together by generic symptoms, as affections pertaining to the same genus, we regard them as possessed of specific properties, by which they are entitled to specific characters, and require specific treatment. When erysipelas appears in a good constitution, uninjured by previous disease; when the circumstances attending its appearance have been favourable to sthenic action, and when, in the symptoms by which it is attended, there is little admixture of debility, it may then be regarded as an affection essentially inflammatory, and will require active treatment; but when it involves parts previously weakened by disease, and involves them *in consequence* of that very weakness, as in anasarcous extremities; or, when it appears in the erratic form, flying like a consuming flame from surface to surface, over a worn-out system, we cannot imagine an excuse for a principle, which teaches the immutable character of erysipelas under all circumstances, its sameness with acute inflammation, and the expediency of a more or less rigid antiphlogistic treatment.

Using erysipelas as the generic term, he observes:—

"When it affects the surface of the skin, which is red, not sensibly swelled, soft, and without vesication, it is called *erythema*. *Simple erysipelas* is a more violent cutaneous inflammation, attended with effusion into the cellular substance, and generally with vesication; *phlegmonous erysipelas* is the highest degree of the affection, involving the cellular and adipose membrane as well as the skin, and causing suppuration and mortification of the former." 2.

In the *simple variety*, the skin is red and shining; the colour, which varies from a rosy tint to a bright scarlet or livid red, disappears on pressure, but soon returns when pressure is withdrawn. The inflamed parts are hot and painful, but the pain is more constant and less acute than in pure inflammation. It is more smarting than lancinating—more ardent than pungent. There is little tumefaction and no tension, until the cellular tissue be involved, when effusion taking place, causes swelling, tension, and a shining surface. This infiltration is seldom, however, confined within the cellular texture, when the erysipelas becomes severe; it more frequently elevates the cuticle into vesicles or bullæ, similar to those produced by cantharides, which are usually of a straw yellow colour, but differ occasionally in appearance, according to the nature of the fluid they contain, which may be either purulent or bloody. When these serous collections arrive at their maximum size, they burst, their contents form crusts which soon exfoliate, and the blistered surface seldom passes into ulceration. Gangrene is a rare occurrence, and if the inflammatory action be equally diffused, suppuration is seldom witnessed.

One of the most peculiarizing symptoms of erysipelas, is its tendency to spread. In consequence of this property it has received its name. Sometimes it moves about from surface to surface, without passing through all its stages in any. To-day it appears upon the back, to-morrow upon the abdomen; now upon the head, then upon the extremities; and while its visits are so numerous, they are generally short.

When is this travelling *penchant* observed in true inflammation? A texture is selected from some predisposing quality, increased action is commenced, and the part which is first exposed to the kindling flame, is usually the last to witness its extinction. But erysipelatous action prefers surface to substance, and the varieties of liberty to the uniformity of confinement.

The constitution almost invariably sympathises with this local action. There are shiverings, alternated with heat, torpitude, head-ach, anorexia, foul tongue, nausea, and costiveness. When the head is the part affected, sleepiness is a common, and coma or delirium not an unfrequent symptom; the tongue is likewise more foul, the strength more depressed, and the pulse less powerful.

The *phlegmonous variety* differs principally from the simple form in the greater intensity of its action, and more violent character of its symptoms. The redness of the integuments is deeper, and often mottled—the tumefaction is greater, and pits slightly upon pressure—the appearance of vesications is uncertain—destruction of the cellular membrane soon begins—the skin becomes livid, and studded with phlyctenæ, and the general symptoms are materially aggravated. When an entire limb suffers, the cellular texture suppurates and sloughs, detaching the skin, penetrating between muscles, inflaming the synovial membranes of contiguous joints, ulcerating their cartilages, producing anchylosis, and occasionally caries of the bones. The absorbent glands are generally enlarged, and the neighbouring lymphatics tender.

"An inflammation of such extent and violence cannot fail to excite the most serious sympathetic affections, among which may be mentioned disturbance of the nervous system, causing symptoms of typhoid character, inflammation of the lungs or pleura, of the intestinal mucous membrane, producing diarrhœa, or of the peritoneum, and inflammation or suppuration of other organs. The combination of the primary and secondary affections is speedily fatal. If, however, the patient should recover after tedious suppurations and discharge of sloughs, the parts, which have been inflamed, are so changed in structure, and skin, fascia, muscles, tendons, and bones are so unnaturally agglutinated and fixed, after the extensive destruction of the connecting cellular texture, that the motions of the part are permanently and seriously impaired." 13.

The skin and cellular texture are the seat of erysipelatous inflammation, but it is a circumstance somewhat singular, that pus is found more frequently in the tela cellulosa adjoining the fasciæ or muscles, than in that which is exterior to the aponeurotic expansion.

"If, which is doubtful, Mr. Hutchison means that the fasciæ or aponeuroses, properly so called, are the seat or principal seat of the disease, I cannot agree with him, having always found them unaffected in examinations after death, and seen no symptoms referrible to such an inflammation during life. They may indeed become involved in the disease, when it is violent; and they must suffer partially when it extends to the intermuscular cellular structure; but they are not primarily affected in these cases, while, in the majority of instances, they do not suffer at all." 16.

But there can be no doubt as to Mr. Hutchison's meaning, for his language is too unequivocal to admit conjecture. Speaking of erysipelas phlegmonodes, he says—"that species of the disease at present under review is, I am persuaded, confined chiefly to membranous parts, such as the aponeurotic expansions, skin, sheaths of tendons, muscles, &c." p. 114, *(Surg. Observat.)*; and although Mr. H. is supported in this assertion by Earle and Arnott, we agree with the author in believing, that the fasciæ are merely involved by consequence, and only in cases unusually severe. The cellular tissue is of a softer and less resisting texture than fascia, which can withstand an action that would be destructive to the former; and it is undeniable that we often find, upon dissection, tendons and aponeuroses entire and unhurt, when the surrounding fat and muscles have wholly disappeared. From the nature and position of the parts subject to erysipelatous action, it must be obvious that any division of this disease, depending for its accuracy upon the individual texture involved, must be more fanciful than just. The skin and subjacent cellular structure are so immediately connected, that the one cannot be long or seriously affected without implicating the other; and when we consider that action does thus pass and repass by insensible shades into contiguous organs, its character in either cannot be very different, and the treatment to be pursued in both must be materially the same.

In accordance with the principle, that erysipelas and inflammation are one, Mr. L. argues strenuously against those who maintain the former to be possessed of specific characters.

"I can however by no means agree with those who regard it as a distinct species of inflammation, and as capable, in that character, of affecting various parts of the body as well as the skin. Some writers have referred to erysipelas certain inflammations of the conjunctiva, mouth, and fauces, of the respiratory and alimentary mucous surfaces, of the serous membranes in the head, chest, and abdomen, and of the brain, abdominal and thoracic viscera. The proof of such an opinion would consist in shewing that the same peculiarities, which distinguish erysipelas from other inflammations of the skin, are found in certain inflammations of the parts just enumerated, and that such affections may hence be distinguished from ordinary inflammations of the same organs." 21.

A priori, we see no conclusive reason why a disease, so partial to superficial structure as erysipelas evidently is, should be confined to the external surface of the body. We know of no prescriptive right by which the external integuments have appropriated this affection, and many acute and experienced observers have recorded instances, without number, of internal parts having betraye d every specific symptom of erysipelatous action. Mr. L. admits that—

"The texture of mucous membranes presents indeed some traits of analogy to that of the skin, and there is a corresponding conformity in some of the morbid phenomena. Thus, so far as organization is concerned, we might suppose that mucous membranes would be susceptible of erysipelatous inflammation; but we see nothing that is clearly referrible to this head, either during life or in examinations after death, although these membranes and the skin exert over each other, in many cases, a powerful sympathetic influence." 22.

If a diffused redness, accompanied with a hot or smarting pain, appearing and disappearing without completing any definite period, or terminating by any marked crisis, be signs of erysipelas, they have been often witnessed in the mouth and fauces; and if these symptoms have appeared upon the subsidence of erysipelas of the face, or have vanished upon its appearance, it must be admitted that we have strong presumptive, if not positive evidence. Cases have been recorded by Dr. Abercrombie, in which the lining membrane of the nostrils formed the medium of communication between the internal and external inflammations; and Dr. Stevenson has furnished us with many histories illustrating the same communicability of action. Some have thought that diarrhœa, puerperal fever, and ophthalmia, often arise from the same cause; and others have gone so far as to maintain that the solid viscera are not exempt from its influence. But, without stretching our theory to such an objectionable length, we must, at least, allow facts, fairly stated and unexceptionably attested, to make that impression, and meet with that respect to which they are entitled. To the absence of vesication, and even of visible inflammation, in parts suspected of this disease when examined after death, we attach no importance; because Mr. L. admits, and every tyro knows that the former is not essential, and that the latter can be seldom noticed even on the external surface, the inflamed parts having returned to their natural colour.

But some are much more restrictive in their views of erysipelas than Mr. Lawrence. Mr. Arnott, for example, would confine it to denote inflammation of the integuments of the face, because he considers that this form of the disease is contagious, runs a specific course, and is attended with sensorial disturbance. The following judicious remarks form a sufficient reply to these objections:—

"The affection of the sensorium and the fever seem to me to arise from the brain participating in the inflammatory excitement of the contiguous and connected parts. Should we not expect à priori that erysipelas of the head would produce much more severe sympathetic effects than that of a limb? Making the necessary allowance for slight difference of structure, the inflammation itself is exactly alike in the two cases; there is no difference in the appearance of the skin during life, nor in the changes observed after death. The facts related and cited by Mr. Arnott lead, on the first view, to a strong suspicion of contagion; but in order to justify on this ground the marked distinction which he has drawn, it would be necessary to show that erysipelas of the face is, and that erysipelas of other parts is not, contagious; neither of which points is hitherto proved." 25.

In settling the nosology of erysipelas, the author is naturally led to examine the arrangements of various writers; and we think that he rejects with justice the classifications of all. Nothing, certainly, can be more confused than Cullen's disposition of the different species of this affection. Erythema he places among the phlegmasiæ, and erysipelas among the exanthemata; dividing two shades of the same action, to be often, if not always, seen more or less together in the same case, into orders, separated by the most distinctive characters. J. P. Frank has placed erythema among the impetigines, and erysipelas among the exanthemata; whereas Willan and Bateman class erythema with the exanthemata, and erysipelas with the bullæ; and, although Rayer has improved upon his predecessors, by ranking them together, he introduces them into the same order with miasm and small-pox, from which they most essentially disagree.

"In a natural nosology, erysipelas would be classed among inflammations of the skin and cellular texture, and would follow erythema, to which we should refer slight superficial and partial inflammation, without vesication, generally without swelling or fever. This slight inflammation may extend into the cellular membrane, and be attended with swelling, as in the erythema nodosum of authors.

"Of erysipelas, which may be called spreading inflammation of a considerable portion of the skin, with diffuse redness and swelling, sometimes preceded and generally accompanied by fever, it would be sufficient to admit three species; namely,—

"1. Erysipelas simplex; superficial spreading inflammation of the skin, with bright scarlet or rosy redness, and soft tumefaction of the part, generally with vesications and fever.

"Synonyma.—*True* or *genuine erysipelas.*—*E. exanthematicum*, or *verum* (Rust, on erysipelas of the face.)

"Varieties.—*Acutum; chronicum; periodicum; habituale; perstans* or *fixum; ambulans* or *erraticum; saltans* or *volaticum* (disappearing from its original seat, and re-appearing in a distant part); *miliare, vesiculosum, bullosum, phlyctenodes; idiopathicum, traumaticum, sympatheticum*, or *symptomaticum, biliosum, gastricum.*

"2. E. Œdematodes; the swollen part dark red, and pitting on pressure.

"3. E. Phlegmonosum; acute inflammation of the skin and cellular texture, with firm, general, and deep red swelling of the affected part, ending quickly in suppuration and sloughing.

"Synonyma.—*Diffuse cellular inflammation* (Earle.) *Inflammation of the cellular texture* (Arnott.) *Diffuse phlegmon* (Baron Dupuytren.) *E. spurium* or *pseudo-erysipelas* (Rust.) *Phlegmon erysipelatosus.*" 36.

We believe that this classification will serve every useful purpose, and, while we cannot esteem erysipelas as pure inflammation, we hesitate not to adopt the arrangement, which inserts it in the list of inflammatory diseases.

The causes of erysipelas are various, but may be divided into such as act directly upon the seat of disease, and such as excite it by sympathies or intermediate agents. Among the direct causes are all external irritants, as heat, cold, blisters, issues, setons, caustics, wounds, more especially those received in dissection, bruises, ulcers, and other local diseases; while improper diet, chylopoietic derangements, and contagion, pertain to the second class.

The nature of a review will not permit us to enter into the question of contagion in erysipelas, or we should feel pleasure in investigating a subject of so much interest. But the almost entire silence of Mr. Lawrence will be our excuse for a few observations.

In Dr. Stevenson's paper on erysipelas, in the 2d volume of the Edinburgh Medico-Chirurgical Transactions, 21 cases are briefly given, almost all of which seem linked to each other in the mutual relations of cause and effect. The mother of a family, who is the subject of the first case, is taken ill on the 26th of October, her son is affected on the 7th of November, the person who attended her on the 23d of the same month, and her daughter becomes ill on the 25th. The sixth case frequently visited No. 5, the 7th No. 6, the 8th No. 5, the 9th and 10th attend No. 7, and the 9th, who went home, communicated the complaint to her family, and her mother died. In a third series, a Mrs. F. became ill on the 12th of August, her son-in-law sickened on the 30th, her sister on the 17th of September, her nephew on the 1st of November, and on the 1st of December her brother was confined. These are followed by six examples occurring among relatives, where unrestrained intercourse had been maintained.

These interesting cases are alluded to by Dr. Gibson, in his paper at the head of this article, as seen by him in company with Dr. Stevenson; and "the appearance and history of which convinced him that the disease, as it then prevailed in Arbroath, was of a contagious nature." p. 94. Dr. Gibson likewise furnishes several cases very similar. An infant son of a gentleman at Montrose was seized with erysipelas of one foot, the mother became affected upon his recovery, his nurse, being seized with symptoms of pneumonia, went home, a distance of four miles, and her father, who had been some days before wounded in the scalp, was attacked with erysipelas of the wound, and died; a sister, living in the same cottage, sickened, and two children were cut off by what *seemed an attack of croup.* After having attended a gentleman who died with erysipelas, the Dr. was called upon to see a maid-servant of the same house. Finding her to have the same disease, she was sent to her mother's. The mother, in about ten days after her daughter's arrival, was seized with violent febrile symptoms and sunk, and another maid-servant, who had removed the bed-clothes after the gentleman's death, complained of heat and uneasiness in her arms and hands. On inspection they were found red and swoln, but the disease did not spread further. The following extract is very conclusive.

"In 1824, Christian Taylor, from the parish of St. Cyrus, was admitted into the infirmary here with abscess of the hand and caries of the bones of one finger. Some days after her admission, the patients in the two beds next to her were seized with erysipelas, and, on inquiry being made, it was ascertained that the suppuration in Taylor's hand had followed an attack of erysipelas, which had been prevalent in that parish. The patients were all removed from that ward, and it was well cleaned, white-washed, and fumigated, Yet, when patients were placed in that ward, the disease again made its appearance, and it was found necessary to remove the whole patients from our little infirmary, and to take every precaution, before the contagion was eradicated." 99.

Were further instances necessary, we might add many from Dr. Wells, Mr. Arnott, and our own experience; but, believing that if any thing can be proved by cases, plain and obvious facts are as good as arguments; and so we leave the reader to draw the only conclusion which, in our opinion, these facts can sanction.

Mr. Lawrence neglects mentioning foul air and the effluvia of putrid matter in his catalogue of causes, and we are convinced that in many cases erysipelas can be traced to this origin. We have seen neglected night-stools occasion severe attacks, and, without entering into any laboured defence of such an opinion, suffice it at present to observe, that we have several reasons for believing hospitals would be less frequently visited with such a scourge, were all the dejections passed in water and removed immediately they were voided, and were the apartments of the sick preserved in a purer state of ventilation and cleanliness. There are so many points of resemblance between erysipelas and hospital gangrene, that some authors consider them species of the same genus; and we well know the influence which a confined and adulterated atmosphere exerts in predisposing to and producing hospital gangrene. Like it erysipelas is frequently constitutional and epidemic, depending upon general causes, and disappearing under general treatment; and, although it is often excited by local circumstances, and yields to local remedies, it is not a rare occurrence to find it arising from polluted air, filth, and negligence. Hence do we see it haunt certain situations, and select certain hospitals for its own, where no peculiar cause is known to operate, save the contaminatedair of a bad locality. St. George's Hospital in London, and the Hôtel Dieu in Paris, afford marked examples of this fact; for it is notorious, that seldom are the wards of either free from cases of erysipelas. Soubere informs us that this disease is so frequent in Siam, that nineteen out of twenty persons are generally attacked by it. In 1716 it was epidemic in Thoulouse; in 1822 in Montrose; and Bromfield says it was epidemic for two years in St. George's Hospital.

With these obvious and well authenticated facts in view, how can we adopt Mr. Lawrence's language, when he says, that there is really *no difference*, as to the causes of erysipelas, between it and other inflammations; p. 36. Who ever said pleurisy was contagious, or hepatitis originating in contaminated air? No; there is something very essentially different in the symptoms, in the causes, in the nature, in the progress, and in the treatment of erysipelas from pure specimens of inflammation. For the sake of simplifying our nosology, let us not confound diseases; for if our diagnosis be unphilosophical, our treatment must be unsafe. Because two affections have a few points in common, we are not to conclude that the portrait of one will faithfully represent the qualities of the other; and, although erysipelas wear family features of the phlegmasiæ, in the redness, heat, pain, and tension that accompany it, it asserts its own individuality by many characteristic lineaments.

We have considered it our duty thus far to animadvert upon some of the principles contained in the first part of this Essay, because, in addition to the intrinsic importance of the disease in question, and the anxiety which we feel to have it better understood, names now-a-days are imposing arguments, and authority is frequently advanced as a substitute for fact. The talents which ornament, and the learning which distinguishes the author of this paper, will, we doubt not, be held by many as sufficient pass-ports for the sentiments it contains; and, as error is more dangerous the more nearly it is made to resemble truth, so doctrines, advanced by genius and advocated with ability, should be considered with a caution proportioned to the adventitious recommendations with which they are introduced. And, as we now pass on to Mr. L.'s observations on the treatment, we may once for all remark, that, seeing we differ somewhat in our pathology, we cannot agree entirely in our therapeutics. Not that we oppose active measures, on the contrary they are often necessary; but because, holding erysipelas to be a modified inflammation, an inflammation as frequently marked by typhous as phlegmonous symptoms, we cannot warrant the adoption of such antiphlogistic measures as are required to subdue synocha. A modified or mixed inflammation requires a modified or mixed plan of cure; and, although we would bleed, and purge, and starve, we would likewise stimulate, strengthen, and support. Phlegmonous erysipelas in an athletic habit will create symptoms widely different from typhoid erysipelas in an old or broken constitution; yet, while we believe that the lancet will be useful in the one and destructive in the other, we would, as a general rule, deprecate the same liberal employment of it, which pure inflammation sanctions, convinced that the patient, who labours under erysipelas of any form, cannot endure the same extent of depletion with him, who is attacked by cephalitis or pneumonia. There is *a something* peculiar to the nature of erysipelas, and whether that peculiarity arise from something in the texture subject to this affection, or exist as an essential to its very being, we pretend not to determine. We have seen that Mr. Arnott regards erysipelas of the face distinct from that occurring in any other part; and, as almost every organ has diseases peculiar to itself, we know of no reason why the skin may not be subject to an inflammatory variety, "sui generis."

Not being the advocates, therefore, of any exclusive system, we cannot sanction the following general language.

"As this affection resembles other inflammations in its causes, symptoms, and effects, so it must be treated on the same principles; that is, on the antiphlogistic plan. Venesection, local bleeding, purging, and low diet are the first measures, to which saline and diaphoretic medicines may be afterwards added. The earlier these means are employed the better: vigorous treatment in the beginning will often cut the attack short, and prevent the disease from spreading beyond its original seat." 40.

We repeat it, that many cases will occur wherein it must prove fatal to adopt vigorous treatment, even in the beginning. The symptoms do not call for it, the system will not allow it, prudence forbids it, and experience will condemn it. But, although this sentence is couched in such general phraseology, the author very prudently modifies the precept it conveys in a subsequent passage.

"I do not mean to recommend that measures equally active, and, in particular, that bleeding, whether general or local, are to be employed in all cases. In young persons, in the robust and those of full habit, in instances where the pulse is full and strong, or when there is head-ache and white tongue, in erysipelas of the head attended with symptoms denoting affection of the sensorium, and more especially in the very beginning of the affection, venesection will be proper, and it may be necessary to bleed largely, to repeat the evacuation, or to follow venesection by local abstraction of blood. Under such circumstances, the other parts of the antiphlogistic plan must be also employed, that is, the alimentary canal should be cleared by an active purgative, which may be followed by salines and antimonials, with the occasional use of milder aperients; and low diet should be enjoined. Nothing can be more different from such a case, than that of an elderly person with a small and feeble pulse, in the advanced stage of the disease. The interval between these extremes is filled by numerous gradations requiring corresponding modifications of treatment. The antiphlogistic plan itself embraces a wide range in point of degree; from blood-letting, local and general, with purging, vomiting, the free use of mercury and antimony, and low diet, to the exhibition of a mild aperient, with some saline medicine. The treatment of erysipelas, like that of any other inflammation, must be modified according to the age, constitution, previous health and habits of the patient and the period of the complaint. In asserting generally that the antiphlogistic treatment is proper, I speak of the beginning of the disease, when the original and proper character of the affection is apparent; and I am decidedly of opinion that, in some shape or degree, such treatment will always be beneficial in that stage. In many instances active antiphlogistic measures aie of the greatest service in lessening the severity both of the local and general symptoms. In others the administration of calomel with aperients, and of diaphoretics with low diet, will be sufficient. When the affection occurs in old and debilitated subjects, the powers of life are soon seriously impaired, and our efforts must be directed rather towards supporting them, than combating the local affection. I have often seen such patients labouring under erysipelas of the face in its advanced stage, with rapid and feeble pulse, dry and brown tongue, recovered, under circumstances apparently desperate, by the free use of bark and wine." 42.

Except in remarkably acute cases, where every attending circumstance is favourable to depletion, we would prefer local to general bleeding. Ten ounces of blood abstracted immediately from the affected part, will generally produce a much greater and more salutary impression than a double quantity drawn from the system at large; with this material advantage in its favour,—that the strength is not unnecessarily spent, nor the stage of convalescence injuriously prolonged. The superiority of such treatment we have frequently witnessed, and, while we would avoid the charge of inconsistency, in using terms, the universality of which we have just disapproved, we fear not the consequences, either to patient or practitioner, when we unqualifiedly recommend topical depletion. The lancet may be safely applied as a local remedy, when it might be fatal if taken up for a general purpose. Even in those typhoid cases, where the pulse is scarcely to be felt, where muttering and delirium prove the extent to which the nervous system suffers, and where wine and bark and nourishment must be used with an unsparing hand, leeches may be applied, and applied with benefit. The general disorder and local disease mostly *play into each others hands*, and while his sinking powers require support, the patient will frequently be improved by such means as are calculated to remove the source of mischief.

From the well known fact that leeches have occasionally induced erysipelas, they have fallen into very general but undeserved disuse in the treatment of this disease. There is certainly nothing essentially irritating in their bites, otherwise this application should be more frequently succeeded by such consequences. They act, like any other exciting cause, by calling forth a disease to which the part is predisposed, and which would, probably, follow the puncture of a lancet as readily as that of a leech. How often do we see erysipelas of the surface on which cupping-glasses have been applied, and its appearance upon the arm after bleeding is not a rare occurrence! Leeches will be found in many cases very beneficial. They may be used where glasses could not, or should not be employed; and, except in very irritable habits, they are generally followed by relief. The erythematous form is that to which they seem best calculated, the inflammation is cuticular, the cellular structure is seldom or slightly affected, and a trifling detraction will be sufficient to effect every useful purpose. Glasses are attended with many serious disadvantages. The size and situation of the parts diseased will often preclude their application, and the pressure with which they act is most painful and injurious. If a small quantity of blood be required it can be drawn by leeches, if a large one, by incisions. Suction is not necessary to procure blood from an erysipelatous surface. The cutaneous vessels are so active that their division merely is necessary; simple incisions are, consequently, quite sufficient, and the violence occasioned both to the feelings of the patient, and to the textures of the part is avoided.

Mr. Lawrence has taken more trouble than was, perhaps, necessary to collect testimony for and against the antiphlogistic plan of treating erysipelas. He enumerates Sydenham, Cullen, the two Franks, Dr. Duncan, Richter, and Vogel, among the first class; and Drs. Fordyce, Wells, Willan, Bateman, Carmichael Smith, Thompson, and Pearson, among the second. But these lists are equal in number, and in reputation so little different from each other, that neither party can claim victory; authority furnishes no certain conclusions, and we must still consult reason and experience for our rules of practice.

"The direct opposition, both in opinion and practice, which the preceding quotations disclose, must appear very extraordinary, and not calculated to increase our confidence in medical doctrines. I see no mode of reconciling the difference. It is true that erysipelas goes through a certain course, and ends by resolution in many cases, not requiring any active treatment. Bark and ammonia would probably not materially alter the character and progress of the affection: it may be reasonably doubted whether these remedies, more especially the former, have so much influence on the system, as we are inclined to believe." 58.

But such conflicting authorities should not have created amazement in one who observes so attentively as Mr. L. Erysipelas does not invariably wear the same symptoms, or permit the same treatment. Difference of sentiment is, therefore, the necessary result of difference of character; and, although something may be attributed, no doubt, to the inefficacy of every curative mean in some cases, the difficulty, we presume, receives the principal part of its explanation from the fact now stated. Celsus would always bleed; yet Avicenna denounced the lancet. Alexander of Tralles confined his treatment to cold water, and Oribasius exhibited antibilious medicines. Yet these authors may have been as successful in their practice as we, who too often regard unanimity as the test of truth.

"Although Desault restricts the use of emetics to what he calls bilious, in contradistinction to phlegmonous erysipelas, I do not consider them sufficient, as the principal means of treatment in many of those cases. They are, however, useful after the abstraction of blood, where the tongue is furred and nausea or sickness is present. When the latter symptoms are not accompanied by much febrile disturbance, the emetic plan may be pursued, and followed by aperients and diaphoretics. The liquor antimonii tartarizati may be given in the dose of half an ounce every half hour, until the stomach is cleared. This may be followed by calomel and aperients.

"Under the like circumstances, that is, when a white tongue and other symptoms of disordered digestive organs remain after the use of evacuations, the local disease continuing, the free use of calomel alone, or combined with James's powder, is very advantageous: it acts copiously on the bowels, cleans the tongue, and improves the state of the stomach. Two, three, or four grains of calomel, either alone, or joined with two or three grains of James's powder, may be given every six hours, for one, two, or three days. The hydrargyrus cum cretâ might be used in the same way." 59.

The condition of the alimentary canal should be carefully watched in erysipelas, for we have long suspected that it arises more frequently from its derangement than the generality of the profession are aware. Excrementitious matter allowed to putrify in the fæcal tube will not only operate as an irritant upon the whole system, but, from the close and constant sympathy which holds between the cuticular and mucous surfaces, may be expected to exert a deleterious influence more immediately upon the skin. Hence the erysipelas *biliosum and gastricum* of many writers. In the concluding stages of putrid fever, where the bowels had been long neglected before assistance was procured, we have seen the most tedious and inveterate forms of this disease; and, although the removal of this irritation by gentle and continued laxatives did not arrest the progress, it certainly diminished the violence of the symptoms.

"When the means now detailed have arrested the inflammation, the patient is probably reduced in strength, and may seem to require the aid of good diet and tonic medicines. Medical practitioners in general are anxious to begin the strengthening plan; they seem to have the fear of debility constantly before their eyes, and lose no time in directing the employment of bark, and recommending animal food with beer or wine. In this way relapses are frequently produced; the inflammation and fever are renewed, further local mischief is caused, and recovery is retarded. When indeed the redness and swelling of the part are gone, when the pulse is quiet, and the tongue clean, that is, when the patient is well, there can be no great objection to the bark. The natural powers of the system quickly restore its strength, when the disease has been stopped by active treatment; in such cases strengthening medicine and regimen are not required. In others they must be used with the greatest caution. Sometimes they are at once obviously injurious; in others they do good at first, but soon cause a return of the complaint if persevered in. It is safest, therefore, to leave them off as soon as the state of the pulse, or of the other symptoms, which have indicated their employment, is changed. The subcarbonate of ammonia is the best medicine in those cases, in which we doubt whether stimuli should be employed or not. It may be given without any risk of reproducing inflammation, while in most cases it is decidedly advantageous. Five, six, or eight grains may be administered in a draught every three, four, or six hours. Dr. Peart and Mr. Wilkinson regard it almost as a specific in scarlet fever and erysipelas.

"Bark comes next in order to the volatile alkali; and the sulphate of quinine is the most eligible form of the remedy. Wine is sometimes necessary; but it should be used very sparingly, and discontinued as soon as the necessity has ceased." 61.

Some parts of the above extract are expressed in the favourite language of our author; but, however ridiculous the tonic plan may appear in the eyes of Mr. L. the success with which it is attended in cases that are without remedy, if an opposite treatment were adopted, proves its suitableness to nature, and furnishes a voucher in its favor superior to the asperity of sarcasm. If the distinguished authors whom we have lately quoted as opposed to active treatment, had *the fear of debility constantly before their eyes*, their rank and respectability will shield them from the imputation of being ignorant of symptoms, and of trembling for "a lion in the way" where there was neither difficulty nor danger. We believe, in opposition to Mr. L. that *when the patient is well there are great objections to the bark*, or, indeed, to any other medicine. Health requires no remedies, and he, who has no ailment to lament, has a fairer chance of being injured than improved by quackery. The sulphate of quinine will be found in many cases a most valuable salt. In this instance we happen to have Mr. L.'s experience and authority against himself.*

Bark and wine improve the secretions, give a firmness to the pulse, and support the strength. They thus act upon the disease by invigorating the general powers; for the pulse becomes slower and more firm, the temperature falls, the tongue cleans, the thirst subsides, better nights are enjoyed, and the mind gradually regains its lost or injured faculties. We believe, however, that stimuli, even in these cases, may be too long, although they can seldom be too soon employed; and we have more than one reason for inculcating with the author the propriety of omitting them, whenever the effect for which they were intended is produced.

A very extensive variety of local applications have been tried. Those who imagined erysipelas to depend upon an acrid state of the fluids, prescribed absorbent powders and demulcent cataplasms; others, believing it to arise from debility, used stimulating ointments and astringent lotions, while a third order of pathologists, to whom Mr. L.'s views would have been more agreeable, advised refrigerating mixtures to abstract heat, and blisters to subdue action. One applied warmth for the same reason that another applied cold—to make his patient comfortable; and he, who hoped to neutralize the acridity of the fluids by absorbent earths, was guided by similar motives with him, who fancied that soothing unguents would render harmless what he thought it was impossible to remove. But the fruitlessness of all such practice is abundantly established by our daily abandonment of old and introduction of new medicaments; and we concur with the author, when we ascribe their principal value to the relief which they occasionally bring to the feelings of the patient.

With this view cold applications are, in general, calculated for the commencement, and warm ones for advanced periods of the disease. The burning heat of the first stage is allayed by the former, while the latter diminish the tension and sensibility which accompany more matured action. Dr. Peart highly recommends a lotion of equal parts of sugar of lead and subcarbonate of ammonia,—ʒj. of each to Oj. of water; and Mr. Wilkinson's testimony confirms its efficacy. Through the advice of a friend it has been tried by us. In one case it certainly hastened desquamation, but the mind became much deranged upon

* See the last six lines of extract in page 369.

the decrease of the inflammation, and, at the close of the attack, we could neither say it had been much alleviated, or abridged.

"The application of blisters to the inflamed surface, employed in France, has been sanctioned by the high authority of Baron Dupuytren. The phlegmonous and the erratic species of the complaint, more particularly the latter, have been thus treated. In a surgical thesis, printed at Paris, the following summary of the treatment, adopted by Baron Dupuytren in erysipelas, is said to have been taken from his clinical lectures:—

"'If the skin be red, tense, dry and hot, with fever (éréthisme général)—if the tongue be dry and red, a blister or an emetic would do no harm—bleedings and leeches are proper. In a bilious constitution, with the tongue covered by a yellow mucous coat, and bitter taste in the mouth, with pains in the head and bilious evacuations, an emeto-cathartic, or the tartrite of antimony in a large quantity of fluid is indicated. When the tongue is moist and slightly red, the skin moderately tense and hot, when there is local inflammation with little general reaction, the erysipelatous phlegmon should be brought to a crisis by suppuration, excited externally, by one or more blisters. If the local inflammation have a languid atonic character without symptoms of sanguineous or bilious plethora, if, in short, the state of debility be well marked, which will happen particularly in old subjects, we must immediately resort to tonics.'

"The author of this thesis observes, that the application of blisters is not safe when the head, chest, or abdomen is affected; that a blister applied to the neck caused delirium and death; and that a patient died on the following day in consequence of one having been placed on the chest. He states, that in the year 1818, these affections generally ended in suppuration, and that the use of blisters was not so advantageous in that year." 63.

They were extensively applied by Thevenin, but as often in the neighbourhood as upon the diseased surface, with the design of diverting elsewhere the peccant humours. Rayer discountenances them, Roche and Sanson recommend them only when the erysipelas is much disposed to spread, in order to detain it in one spot. Dr. Duncan speaks rather favourably of them, and has never known them to occasion gangrene. In his case of Morand they were decidedly useful. Mr. Hutchison has seen the progress of one case arrested by encircling with a blister the affected limb, just above the seat of action; and Mr. Lawrence has tried them three or four times, in simple erysipelas of the extremities, with the effect of stopping the march of the inflammation; but, having used other means at the same time, he could not determine how much value belonged to them. They did not, however, produce suppuration, or any other unpleasant consequence.

Compression has been employed by some, especially the French, but with such different results that the value of the treatment cannot as yet be estimated. In France it has been highly spoken of; in British practice it has not answered the expectations which the eulogy of Velpeau had excited. But, while we do not think highly of such an application, we apprehend that some explanation for our want of success may be drawn from those cases wherein it has been followed by a fatal termination. In Dr. Nelson's case, as given by Dr. Duncan, the inflammation was more of the phlegmonous than of the erysipelatous kind. The patient, who was a country servant-maid, of middle age, was in a *very strong fever*, *the pulse remarkably strong, and the tongue very foul.* As might have been anticipated the bandage produced excruciating pain soon after it was applied, the arm *became much swoln and inflamed*, several vesicles containing a dark coloured fluid had arisen, her mind was greatly depressed, and she died on the second morning after. Now, we do think that to compress parts in such an active state of phlegmonous inflammation was highly injudicious, and could not have been succeeded by less forbidding consequences. The lancet, not the roller, was the proper remedy. Again, in Mr. Lawrence's case, the patient presented symptoms of a very unfavourable character before the bandage was applied, but there can be no doubt that his death ought to be directly referred to the treatment. He was between 50 and 60 years of age, of a spare habit, and much broken down by misfortune and disease. His pulse was easily compressed, his features sharp and anxious, the integuments of the affected part were of a dusky red, and pitted under pressure; the disease not merely occupied the entire arm, but covered the posterio-lateral part of the trunk, and his mind had been confused the night before Mr. L. saw him. Of course, under such circumstances, active treatment was to be prescribed, and it appeared to the author, *a fair case* for trying the bandage. Through some misunderstanding or neglect, on the part of the attendants, the roller was allowed to remain upon the limb, contrary to the directions left them—

"A period of about twelve hours, although severe pain had come on within two hours from its application, gradually increasing in intensity, with burning heat of the part, and the greatest restlessness, so that the patient endured greater agony than he had ever experienced, and passed a most dreadful night. We found the limb in a condition of universal vesication from the fingers to the shoulder joint, as if its whole surface had been covered with a blistering plaster, which had acted with the greatest effect. One large bladder covered the back of the hand; there were other similar ones on the fore-arm and arm, and all the intervening cuticle was separated from the skin by a thick yellow jelly. A large bladder in the middle of the fore-arm contained bloody serum, and the subjacent cutis was livid. Over the entire surface of the arm, fore-arm, back of the hand and fingers, not a particle of sound adherent cuticle was to be seen. In short, the bandage had acted as the most violent local irritant, and had decided the chances already much against the patient." 197.

To remedy, if possible, this oversight, an incision was made through the skin and adipose substance for *nearly the length of the fore-arm*, from which about 20 ounces of blood were lost, and great relief obtained to the feelings of the patient; but he gradually sunk, and expired in the evening.

It is obvious that no conclusion can be drawn from this case, as to the propriety of bandaging erysipelatous parts. The gross neglect of the attendants, aided by the enfeebled state of the patient's health, is more than sufficient to account for the result, and we only wonder that 12 hours torture occasioned by the roller left surgery a second opportunity of attempting relief. If bandages are to be employed, let them be tried in cases where œdema and other symptoms of local debility are apparent. Pressure may, perhaps, in such instances, assist absorption, prevent the extension of disease, and give an artificial tone to parts which require support. But, in patients labouring under active inflammation, and in constitutional sinking under accumulated misery, we should expect nothing good from a treatment that promises nothing.

In phlegmonous erysipelas depletion may be carried farther than in the simple, or œdematous varieties. The symptoms approach more nearly to those of pure inflammation, and their greater activity requires more active measures. Yet it should never be forgotten that, as a general rule, the local abstraction of blood is more safe than venesection, and not less serviceable. But,—

"If we do not cut the disease short in its earliest stage by this kind of treatment, or if, as very frequently happens, we do not see the case until the inflammation is fully established, perseverance in direct depletion is of little avail in checking its further progress. The inflammation will now pursue its course, both in the cellular membrane and skin, in spite of bleeding, whether general or local; suppuration and sloughing speedily supervene, and these destructive processes soon extend over a large portion of a limb. Indeed I have found that venesection exerts but little influence over inflammation of the cellular texture, and therefore recommend its occasional employment, not so much with the view of arresting the local disorder, as on account of the feverish symptoms when they are considerable." 67.

We have now arrived at that part of the Essay, in which Mr. L. introduces to the notice of his readers a plan of cure in phlegmonous erysipelas, which has lately excited much interest, and which it was partly the object of this paper to recommend. It claims not the novelty of originality, as we shall afterwards more fully show, but its bold and heroic aspect, and the success with which its employment is said to have been attended, give it an intrinsic importance, that needs not to be enhanced by the celebrity of the name under whose auspices it has been revived.

"The most powerful means of arresting the complaint, is by making incisions through the inflamed skin and the subjacent adipose and cellular textures, which are the seat of disease. These incisions are followed very quickly, and sometimes almost instantaneously, by relief and cessation of the pain and tension; and this alleviation of the local suffering is accompanied by a corresponding interruption of the inflammation, whether it be in the stage of effusion, or in the more advanced period of suppuration and sloughing. The redness of the skin is visibly diminished during the flow of blood from the incisions; in twenty-four hours it has usually disappeared, and the skin itself is found wrinkled from the diminution of the general inflammatory tension. The immediate relief, although very desirable to the patient, is however of less consequence than the decided influence of the practice in preventing the further progress of the disorder; and this important result has never failed to occur, within my experience, when the case has been a proper one for the practice, and the state of the patient has admitted of its being fairly tried. The cases already referred to furnish the clearest evidence on these points."

These cases our limits allow us not to detail, but the importance and novelty of the subject will sanction us in grouping into a condensed form their most prominent features, that our readers may be entitled to decide for themselves upon the merits of the practice which they are intended to illustrate.

Of the 38 cases, which are appended to Mr. L.'s observations, 19 were treated by venesection, leeches, aperients, and such incidental medicines as symptoms required; of these none proved fatal. The 36th is the case in which the bandage was applied; the 37th and 38th are examples of inflammation of the cellular membrane, for which general and local bleeding and aperients were prescribed, the first of which terminated fatally;—and the remaining 16 are adduced as illustrations of the incisive treatment. The first 12 of these 16, which he alludes to in the above extract, recovered; the other four, two of which were communicated by Mr. Bullin, perished.

The *first two* of the 12 successful cases are examples of erysipelas of the face in young and healthy subjects, with high inflammatory fever, which was partly overcome by venesection before incisions were had recourse to. The eyelids were the parts incised, and the incisions, which were made transversely, "through the entire depth of the inflamed and swollen cellular structure," gave exit to some fluid and sloughs, after which the parts granulated kindly, and were soon healed; p. 132—135. In the *four* following cases the disease had attacked the arm. The first arose from venesection, the second from an injury inflicted on the elbow, the third succeeded an attack of fever, and the fourth supervened on amputation of a finger. Venesection was employed in three of them before the operation, and the fourth, which is communicated by Dr. Tweedie, from the journals of the London Fever Hospital, had been considerably reduced by an attack of fever to which the affection of the arm succeeded. In one of these cases the incisions were from 10 to 12 inches long; and on that of Cassan Mr. L. observes,—

"This patient was in great danger when the incisions were made; indeed, most of those who saw him considered his situation hopeless. I feared that he might sink under the loss of blood consequent on the incisions, and therefore made only one cut in the first instance (on the 21st of May,) which produced but little relief. Very decided benefit resulted from the two incisions made on the following day, and the more free discharge of blood which they occasioned. This case likewise shews that the affection cannot be stopped by venesection, and that leeches, even when very freely employed, are comparatively inefficacious." 149.

In the *seventh* case nothing particular occurs; the leg and thigh were the parts affected. In the *eighth*, an incision was made through the skin and cellular tissue, nearly the whole length of the right leg, which was followed by about 40 ounces of blood and some pus. A bad night was passed, and on the day following the pulse was very slow and feeble; but, by the judicious use of ammonia, wine, bark, and acids, the strength returned, the wound gradually healed, and the patient was finally dismissed in perfect health. The *ninth* case is very remarkable, and strongly illustrates the virtue of incisions in circumstances the most desperate. The whole foot, leg, and thigh, were *enormously* swollen, and the inflammation extended to the very groin. The lower half of the thigh and the entire leg were covered with vesications;— the pulse was weak and frequent, and the patient was six or seven months advanced in pregnancy; an incision was made *from the ham to the heel*; 24 ounces of blood were collected, some pus was discharged, and the entire limb was covered with a bread-poultice. The next day presented a very different train of symptoms. The inflammation was much and generally reduced, the tension and pain lessened, and a comfortable night had been passed. The sulph. quin. was ordered from weakness having been complained of, and she left the hospital in less than three weeks after the operation, with a sound limb and renovated health. Mr. L. very justly remarks, that—

"This is the most formidable case of phlegmonous erysipelas that I have seen, in reference both to the intensity and extent of the inflammation. When I contemplated the immense swelling of the entire limb up to the groin, with the vivid redness and vesications of the skin, the acute pain, and the firm feel of the swelling, and considered further the advanced state of pregnancy, I apprehended that the termination would be fatal whatever treatment was adopted. The speedy and effectual relief afforded by the incision, and the rapid recovery from a condition of so much danger, illustrate very strikingly the advantages of this treatment. Under any other course of proceedings I feel convinced, either that the patient would have died from the immediate extension of the local and general disturbance, or have been exposed to nearly equal danger from extensive suppuration and sloughing." 159.

Phlegmonous erysipelas of the leg and thigh forms the subject of the *tenth* case; but, excepting the activity with which the disease advanced, there is nothing in its detail very particular. Although 30 ounces were lost by the operation, the lancet was twice required afterwards, and the blood abstracted was exceedingly inflamed. The patient was two months confined before he could use his limb, and even then he was obliged to go with crutches. The *eleventh* case merits particular notice, being one of those formidable phlegmonoid affections which frequently result from dissection. The patient was Dr. Dill, who communicates the case, and a perusal of the symptoms under which he laboured will strike the reader with their similarity to those detailed in Dr. Duncan's valuable paper on Diffused Cellular Inflammation. Two particular features in the history are—that no unpleasant consequences appeared until the evening of the second day after the inspection of the body, and that, so soon as local action commenced, the constitution became immediately implicated, and the disease hastened forward with astonishing rapidity. The incision appears evidently to have arrested the inflammation, and the suddenness with which the symptoms vanished after the operation, was only equalled by the rapidity of their progress before it. Dr. Duncan thinks that the deep puncture which was made in Mr. Blythe's side, was the eventual means of saving his life; and had incisions been early employed in the cases of Mr. Young and Dr. Dewar, we have no reason to suspect that similar success would not have crowned the treatment; for, with Dr. Dill, we are convinced "it may be used, both with safety and effect, in a class of accidents, whose fatal character has hitherto ranked them in the list of our most frightful diseases." p. 169. As the last of the twelve successful cases is very similar to the former in its nature and treatment, we will pass on to give a more detailed account of the four which ended fatally.

The subject of the first was Charles Neale, aged 47, who was seized with phlegmonous erysipelas of the fore-arm, in consequence of a wound inflicted on it by himself while making an attempt upon his life. He first cut his arm, and then made a large deep wound between the thyroid cartilage and hyoid bone, which laid bare the carotid artery, without dividing any important vessel. Forty-eight leeches having been applied without relief, two incisions were made through the skin and cellular membrane of the fore-arm, extending nearly *the length of the limb.*

"Blood flowed from these at first rather freely, but not more so than is desirable for procuring relief to the inflamed and distended parts: the bleeding gradually stopped, and had ceased in about three quarters of an hour, when the patient fainted; before any means could be adopted for his restoration, the state of syncope had terminated in death. As the arm had been laid in a broad wooden platter, the blood was collected, and afterwards measured; it amounted to a little more than twenty ounces." 175.

Post-mortem examination gave no account of the fatal event. The body appeared stout and robust—the incisions had not penetrated the fascia—a large transverse opening was found in the larynx, between the glottis and epiglottis, but without injury of either—some water lay beneath the arachnoid and in the ventricles—much of the serous membrane of the lungs was cartilaginous, and beneath it was a thin stratum of compact matter, as black as soot. The condition of the abdominal viscera is not noticed.

"I think (says Mr. L.) we must ascribe the principal cause of death, in this instance, to the mental depression and constitutional derangement which preceded and accompanied the erysipelas. In a man so robust, it is difficult to believe that the loss of 20 ounces could of itself produce death; the despondency which urged him to suicide, and the depression which forbade all hope of recovery, were strong predisposing causes; the wound in the larynx increased the general irritation, and the hæmorrhage, so instantaneously produced, made an impression upon the system for which it was badly prepared, and accelerated an event that would, in all probability, have occurred without the aid of the scalpel."

In the *second* fatal instance, a man, of an unhealthy appearance, was attacked with erysipelas œdematodes of the foot and leg, from a severe blow upon the ankle. Sixty leeches, fomentation and poultices were applied, and 14 ounces were taken from the arm, which had the effect of procuring some temporary relief at much constitutional expense, for his pulse became feeble, his countenance sallow, and his tongue thickly coated. He was now ordered 10 grains of subcarb. ammon. every six hours, by which his general symptoms were for a time improved, and the redness and swelling of the leg diminished.

"The tongue, however, did not become clean, and an inactivity and even drowsiness, which had been observable from the time of admission, were not lessened. On the 19th there was considerable inflammation on the outside of the foot, which was much swoln, tense, and acutely painful. The tongue began to show a slight brown tint, and was rather dry; he was thirsty, and inclined to doze. Mr. Lloyd and other gentlemen recommended a free incision under the supposition that matter had formed; and, as I considered the measure advisable, although I could not distinguish fluctuation, I made a cut about two inches long through the skin and cellular texture, which was loaded with serous effusion. The wound bled so little at the moment, that sponging with tepid water was directed to encourage the bleeding, which soon became more free. The nurse, to whom this duty was entrusted in consequence of the sister of the ward being ill in bed, allowed the blood to flow for nearly two hours, before she apprised the house-surgeon, who found the patient with a very feeble pulse and cold extremities. By raising the limb, and placing a bit of lint between the edges of the wound, the hæmorrhage was immediately stopped. The pulse rose, and the heat of the body was restored by the administration of wine, brandy, and ammonia. The local inflammation was completely relieved, and the erysipelas disappeared entirely from the leg. Wine and stimulants were continued, and the pulse regained its strength. The tongue however was rather dry, and covered with a yellow crust in the middle, the discharges from the bowels were unhealthy, and the listlessness with disposition to sleep remained the same. This kind of sensorial oppression increased, the tongue became brown and quite dry, and he died on the 28th." 180.

The *third* case is that of a gentleman, whose glands above the inner condyle of the right arm became inflamed, swoln, and rather painful, the morning after having examined the body of a person who had died of phthisis. When Mr. Bullin, who gives the history of the case, saw him, he had much head-ach, sense of oppression at the præcordia, hot skin, yellow tongue, flushed face, and rather dilated pupils. His pulse was 90, soft, and his bowels regular. Twelve ounces were taken from the nape by cupping, 12 leeches were applied to the arm, and a brisk purge given. A slight remission was the result of this treatment, but in the evening, head-ach having returned, 24 leeches were put upon the temples, the hair removed, cold wash applied, and three grains of extr. hyoscyam. ordered at bed-time. On the 24th, (the next day) the head was relieved, but the arm was worse. Six leeches to arm, afterwards 10 ounces by the lancet, other medicines continued. *25th.* Symptoms more favourable, but still heat and pain of head. Pulse 120, soft; 20 leeches to temples, and, at night, venesection to 20 ounces, which caused syncope. *26th.* Arm enlarged, and erysipelas extended to shoulders: pulse 120, soft; 12 leeches applied. *27th.* Symptoms unchanged, except increase of head-ach; blood from the nape, and 12 leeches to the temples. On the 28th, Mr. L. saw him, and made two incisions in the inflamed parts—one in the arm, the other in the fore-arm, from which two or three pounds of blood were lost, when syncope came on. The operation was followed by dimness of sight and feeling of alarm; but his head became cold and free from pain—the arm was less red and swoln, and the pulse fell to 96. *March 1st.* No sleep, although two grains of opium had been taken, and the mind had wandered during night. Some hiccough, and respiration laboured, but the arm continues better. Wine, ammonia, and opium are now given during day, and an enema of opium and assafœtida at night. On the morning of the second he died. Mr. L. commenting on the above interesting history, observes:—

"It is clear from the narrative of Mr. Bullin, that the progress of the local mischief was arrested by the incisions, and that they produced, what all the previous measures had failed to effect, a very marked diminution of the inflammation. Whether the fatal event happened in consequence or in spite of them, is a question on which opinions will differ, and light would probably have been thrown by examination of the body.

"It does not appear to me that Mr. B. died from loss of blood. It has been suggested that he perished from the shock of the operation. If we understand by that phrase a violent impression on the nervous system, ending fatally within a short time, the explanation is hardly admissible in this instance. I remained in the room, after making the incisions, about ten minutes, during which nothing occurred to attract particular attention. At the end of twenty-four hours, when I again saw Mr. B. he was sleeping tranquilly, with a steady and quiet pulse, by no means deficient in strength; and the state of the arm was greatly improved. He died thirty-six hours after the incisions had been made.

"When we consider how often death has ensued from wounds received in dissection under various plans of treatment, we need not be surprized at a fresh instance of fatal termination. When we reflect further that both the local and general symptoms were severe in this case, and that, at the time of making the incisions, the inflammation not only occupied the whole upper extremity, but had even extended to the body, we may reasonably doubt whether the event of the case was in any way owing to that treatment." 188.

A young lady is the subject of the last case. An abscess had formed over the first joint of the great toe, which discharged some pus when opened with a lancet, which gave very immediate relief. Shortly after, however, a rigor and head-ach came on, lancinating pains from the toe to the groin were complained of, and syncope supervened. Fomentations, poultices, and a brisk cathartic, were ordered, but, on the next day, all the symptoms were much aggravated, and the patient lay in a high state of fever. A patch of inflammation was now visible about half an inch from the injured joint, and the groin, and even axilla, were painful; eight leeches to temples, six to foot, other medicines continued. 2d. Toe quite relieved, and head somewhat easier; 12 leeches to temples. Repet. alia. On the 4th two other spots appeared, for which 10 leeches were applied, the other remedies repeated, and opium ordered at night. *5th.* Foot better, but increased constitutional disturbance. Mr. L. saw her on the 9th, when she was found labouring under phlegmonous erysipelas of the foot and leg, and slight symptomatic fever. The countenance was rather wild, the features sharp, and the pulse vibrated strongly. "Two incisions, of three or four inches each, were made, one in the foot, the other in the lower part of the leg, and a small quantity of blood flowed from them." A slight improvement followed, but the fever soon returned, and the pulse never lost that vibratory character, which the author considers very unfavourable. She died on the 23d, after every variety of general and local treatment had been employed, although the inflammation never passed the knee, and the redness and swelling had disappeared for three or four days before death.

"The latter event was caused by a continuance of the feverish disturbance, which was attended throughout by the vibratory pulse already described. Venesection was twice practised in small quantity, with partial relief; the blood being strongly buffed and cupped. The pulse ranged from 120 to 130—it was occasionally higher and lower; once or twice it was so low as 108, but never less. The head was shaved, and covered with cold wash; it was not affected in the earlier period of the complaint; but delirium came on, first at night, and afterwards through the day also. Salines with antimony, purgatives, calomel and digitalis, were all fully tried, with only temporary benefit. That nothing might be omitted, we employed hyosciamus and opium in the latter stage, and gave a trial to tonics and cordials, sulphate of quinine and porter being the articles selected for this purspose. They were used most cautiously, but were decidedly injurious. In this case neither the erysipelas nor the incisions were the cause of death, which must be ascribed to the fever, originally excited by local irritation, kept up and aggravated by the free use of opium, in the beginning of the affection." 193.

The above short *digest* of Mr. L.'s treatment, with its results, will, we hope, enable the reader to form his own estimate of its importance. Many of the cases in which incisions were practised, were unusually severe, and, when compared with the first 19 that were managed in the ordinary manner, presented very little prospect of recovery. It is true that none of the first proved fatal, and that many of them were more quickly dismissed; but the mortality which occurred in the latter number was a small proportion of the cases treated, and when we include in our calculation the inveteracy of the symptoms, and the diseased habits of many of the subjects upon whom the operation was performed, we must ascribe to the efficacy of the treatment not a few of the recoveries, while we can easily discover, in some of the cases which perished, sufficient cause of death in circumstances independent of the operation. The fatal termination of the *first* and *last* cases cannot, we think, be attributed to the treatment; our comments upon the first we have already given, and with Mr. Lawrence's explanation of the last we entirely concur. The *second* and *third* are those alone which may give rise to difference; but as to the *third*, Dr. Duncan's paper is suffieient to prove the fatality of such accidents under any plan of management. The only question which occurs to us is, whether incisive treatment be judicious in cases so desperate and far advanced.

The grand object of incisions should be the *prevention* rather than the *removal* of the effects of inflammation; or, in other words, they should give exit to blood, rather than to pus and sloughs. We cannot agree with those who advocate the employment of extensive incisions in the closing stages of inflammatory action. When pus is formed, it is unquestionably proper to remove it, but let it be removed at the smallest expense to the constitution. To make furrows in the integument, of 10 and 14 inches, under such symptoms, we hold to be unnecessary in its object, and dangerous in its tendency. The period of depletion has then generally passed; the time for support has generally arrived. The good to be expected from such lengthened incisions cannot be procured, because the parts and the symptoms are not in that state in which they are required. It is when the action is beginning, has commenced, or is at its acmé; when the heat is burning, the thirst ardent, the tension great, the pulse strong, the pain acute, and the texture full of blood—that the constitution, so artificially excited, can support, and the integuments, so plethoric, can require such incisions. Then the scalpel will disemburthen the gorged integuments of their oppressive load, will restore the distended vessels to their wonted size, will subdue the activity and reduce the temperature of the stimulated textures, and will prevent the approach, or curtail the progress, of that matured action, which only finds its cure in the secretions which it forms. But if we wait until the disease has crept among the tendons and the fasciæ; until the cellular tissue have died; until pus travels, by every outlet, from the original seat of action over the entire limb, and, by extending the excitement, propagates the mischief to an indefinite degree;— we wait until our 14 inches should be reduced to four, and until our object is as much to remove the proceeds of past action, as to restrict the progress of what we have to dread. We have witnessed, and we have practised, and we are friendly to Mr. L.'s treatment, but we would urgently advise its adoption at an early period, in fact, so soon as the disease is recognized. From all that we have seen and read, we can discover no argument for delaying, but we know of many for immediately employing the scalpel.

The truth of these observations is well illustrated in several of the foregoing cases, particularly the 5th, 7th, 9th, 11th, and 12th. Excepting in the 9th, no pus had formed before the operation; the cellular membrane was merely inflamed, firm, and full of blood, and the recovery was uninterrupted and rapid. Whereas, in the four fatal cases, the operation was long delayed, and the cellular membrane was found dead and sloughy in almost all those patients whose convalescence was protracted. We, therefore, wish Mr. L. had insisted at greater length upon a point which must be admitted to be of much importance; for, although in one or two scattered sentences, the reader is led to understand that the incisive treatment is most effectual in the first stage, the fact of its being so is not held forward in that prominent manner to which a leading principle is entitled.

After observing that he does not advise incisions in erysipelas generally, but that he confines their employment to phlegmonous cases, he very justly adds—

"We cannot however determine our treatment merely by reference to the name of the affection. Diseases appear quite distinct in nosologies, but we find them shaded off and so blended in nature, that it is frequently very difficult to mark out their boundaries. The presence or absence of inflammation of the cellular texture will not afford the criterion we are in search of on this occasion; indeed the difference between simple and phlegmonous erysipelas is rather in the degree than in the seat of the affection. Simple erysipelas seldom takes place without some inflammation of the cellular membrane. We may trust to the antiphlogistic treatment already described in doubtful cases, in the milder instances of phlegmonous erysipelas, and in the very early period of the more severe ones. It is important, however, to decide the point quickly, and to make the incisions without delay where we judge them necessary, as they stop the progress of the disorder, and prevent the occurrence of suppuration and sloughing." 70.

It is in phlegmonous erysipelas of the limbs that they are most effectual; in that of the face, Mr. L. does not recommend them, believing that it has a tolerably regular course, ending either in resolution, vesication, or desquamation, and that it may be treated as simple erysipelas. In that of the head, when it is exterior to the aponeurosis, they are not necessary; but when it invades the cellular tissue under it, early incisions will prevent the formation of matter, and much pain to the patient.

But their employment need not be restricted to purely phlegmonous erysipelas, for we have seen, in the cases of Dr. Dill and Mr. B. that they have been extended to a most important class of accidents, and, although in the latter with a fatal result, had the scalpel been taken up as early as it was had recourse to in the former, we doubt not the patient would have had a better chance of recovery.

Much has been said about the length to which the incisions should be carried, and much unjust and unnecessary ridicule has been thrown upon the practice, from the belief that Mr. L. indiscriminately recommends long incisions. The groundlessness of such a rumour the following passage will sufficiently expose—

"As the numerous short cuts, or the single longer incision will equally answer the end, the selection may be left in each case to the patient or the surgeon. The incision should divide the skin and the cellular texture down to the fascia; it is not necessary to penetrate the latter. A double-edged bistoury is the most convenient instrument for the purpose." 78.

Some, as Mr. Guthrie, recommend them to be long; others, as Mr. Hutchison, advise them to be short; and a third class of practitioners, as Dr. Dobson, while they approve of, and act upon, the same principle, are satisfied with mere punctures. But, we believe, no general rule will universally apply. The disease may be so extended, the constitution so robust, and the system so plethoric, that incisions of ten, or even more inches, may be necessary; whereas, cases may occur in which punctures would be the best, because the safest mode of giving relief to the distended, but weakened texture.

As the cuts are sometimes followed by profuse hæmorrhage in weakened and old people, we must be cautious, and not allow it to proceed too far. To arrest it, it will, in general, be found sufficient to elevate the incised limb, and apply pressure; but if these means fail, a ligature will be necessary. If the bleeding, on the other hand, have stopped too soon, cloths wrung out of warm water will encourage it, and when a proper quantity of blood has been obtained, the incisions should be covered with a warm bread poultice. They soon begin to suppurate, and if nothing occur to impede the cure, they granulate quickly, and soon fill up. When the scalpel is used in a late stage, the suppuration is much more copious, the cellular membrane sloughs away, and the skin is detached to a considerable distance around the sore. In these cases the waste is often great, and the strength requires support.

We have now closed a full and, we trust, an impartial review of a long and valuable paper; and, while in some points neither our pathology nor experience corresponds with the sentiments it inculcates, we have differed with reluctance when opposition was called for, and we have been careful to acquaint the reader with the reasons upon which our opinions have been formed. Perfect unanimity in medicine will never be enjoyed, and, perhaps, it is not to be desired. Difference of judgment elicits truths that would be otherwise unknown; and although disagreement implies error, concord is not necessarily the proof of truth.

Mr. Lawrence does not, as we before observed, claim the merit of originality in recommending this plan of treating phlegmonous erysipelas. O'Halloran was probably the first who taught it, but he confined it to a species of gangrene subsequent to phlebotomy. It was afterwards mentioned by Pott and Vogel; Dr. Johnson, in 1814,* recommended it strongly in erysipelas of the extremities; Mr. Hutchison, in the same year, published a paper upon the subject, and it has been variously noticed by many subsequent writers. But Mr. Lawrence has certainly investigated more largely than his predecessors the claim which it has upon the profession; and, while the public will be able to appreciate the talents with which this investigation has been prosecuted, they must feel indebted to the author for the perfection to which his conclusions have been brought.

---

Immediately succeeding Mr. Lawrence's paper, which occupies 205 octavo pages, we have two short communications from Dr. Dobson, of Greenwich Hospital, and Mr. Copland Hutchison. Some particulars of the former paper were given by us at the time it was read, and will be found at page 514 of our 16th number for April, 1828. The following extract will convey the substance of Dr. Dobson's paper in his own words.

" 'With regard to the nature of erysipelas in which I use the punctures, I answer in all cases, whether simple, traumatic, or phlegmonous; the number of punctures

---

* Report from the Scheldt fleet, New Medical and Physical Journal, May, 1814. Dr. J. claims not the merit of discovery, but merely the priority of publication on the subject.—*Ed.*

I make at any one time varies according to the extent of the disease, but is rarely under ten, and seldom exceeding fifty; the depth and extent of each puncture vary also according to circumstances, being made deeper when the parts are more tumid, but more superficial when the tumefaction is not so great; from two to four-tenths of an inch may however be considered the proper answer to that part of your inquiry. I repeat the punctures to the number and extent required, mostly twice a-day, and often in bad cases three or four times in the twenty-four hours, and in the whole course of this practice, whieh has been resorted to by me in several hundred cases, having adopted it more than a dozen years ago, I have never seen any bad consequence resulting from its employment. The quantity of fluid (for it is not blood alone, but blood and effused serum) which these punctures discharge, although sometimes considerable, need never create any alarm, for however freely it may flow at first, it gradually diminishes and soon spontaneously ceases. I use these punctures in every part of the scalp, or face, body, or extremities, and never more freely than about the eyelids, and I have often found a patient with both eyes closed, which by freely puncturing he has been able to open in a few minutes; and what will be found not less true, than it may appear surprising, these punctures mostly heal in a few hours, and never entail any material marks upon the patient!

“ ‘Where puncturing has been practised from the first appearance of the disease, suppuration rarely takes place, and I have observed that it always diminishes the extent of that result even in those cases which have existed for some days before it has been resorted to: but when matter does at any time form under the skin, I let it out without delay wherever I feel it, but I think the integuments in those cases are more preserved by making several small openings, than by one large incision, and the matter is quite as well evacuated. And I am perfectly sure that before suppuration, puncturing, which can be repeated again and again as occasion may require, has every advantage over large openings, which, like punctures, cease to bleed before the disease is subdued, but which cannot like them be renewed, and are often followed by extensive ulceration.

“ ‘The adjuvants which I use with the punctures in this disease are in the first place a brisk cathartic of extract of colocynth, scammony, and calomel; I then prescribe the following mixture, which, while it keeps up the free evacuation of the bowels, acts rather cordially upon the stomach:

℞. Mistur. Camphor, f℥iii. Liq. Ammon. Acet. Tincturæ Rhœi āā f℥iss. M.
Sumat cochlearia duo larga 3tiâ vel 4tâ quâque horâ.

I also employ a lotion composed as follows, viz.

Liq. Ammon. Acet. Oss. Sp. Camphor. f℥j. Aquæ puræ f℥vij. M.

Which, however unchemical it may seem from the camphor and spirit being divorced, experience has proved to be both a beneficial and a comfortable application, and may be always used with the punctures without any fear of revulsion or metastasis, which have sometimes resulted from the use of cold lotions without local abstraction; and it is no uncommon practice for me to prescribe wine or even gin for my patients in this disease at this establishment, at the very time I am puncturing them twice or thrice a day!” 209.

---

Mr. Hutchison's communication consists of a case and a few remarks on the practice of incisions generally.

The subject of this case was a man, aged 52 years, of spare and relaxed habit, who had received a slight abrasion on the ridge of the right tibia, from the iron step of a gig. This was neglected; and when Mr. H. saw the case, there was only “ a *slight* degree of erysipelatous inflammation, of the *erratic* species, and of a red brick-dust colour,” and extending from the tuberosity to within four or five inches of the ankle. Towards the centre of the gastrocnemius there was a very painful spot. The treatment had been sulphate of quinine and wine, with leeches to the inflamed part, and fomentations. This treatment was approved of, and continued; but, next day, Mr. H. was startled “at the appearance of a mortified patch of integument, the size of a dollar, on the outside of the leg, the patient's pulse at this time continuing of the same strength and frequency." Mr. H. now made six or seven incisions down to the muscles, each an inch in length—two into the sphacelated part. In six hours the sphacelus was arrested—the pain greatly diminished—and matter exuding from the incised parts. There was a subsequent demonstration of sphacelus, which was stopped by a single incision. The patient did well.

In Mr. Hutchison's "remarks," he queries whether, among the crowded inhabitants of London, where erysipelas is occasionally so insidious in its attacks, and dangerous in its issue, "it might not, in the great majority of instances, eventually prove to be the best and safest practice to have recourse to incision at once." We have a notion that, in private practice, this cannot be done, whether it be the safer mode of treatment or not. Mr. H. suspects that Dr. Dobson's plan will not prove effectual in ERYSIPELAS PHLEGMONOIDES, where the seat of the inflammation is deeper than the cutaneous tissues—and we are inclined to agree with him. From Dr. Dobson Mr. H. turns to his friend Mr. Lawrence; but as we are not very eager to come between two combatants armed with keen scalpels, we shall let Mr. H. speak for himself.

"I do loudly protest against the practice of making the incisions of such length as recommended by Mr. Lawrence, both as unnecessary, and not so likely to stop the progress of the disease where it is spread over a wide surface, as several smaller incisions made on different parts, where the disease is found to be most active; for it will have been seen, by the closely observing surgeon, that when this disease runs on to suppuration or to gangrene, for example, abscesses or gangrenous patches are occasionally found to have taken place in different parts laterally distant, and having no communication with each other. Now if one long incision be made in a direct line through the middle of the inflamed surface, according to Mr. Lawrence, the disease may be still unsubdued, though greatly lessened on each side of it, to a certain distance. But supposing the disease be found to occupy a space from the great trochanter to the toes, including the whole circle of the thigh, leg, and foot, which I have witnessed in two or three cases, wherein eighteen incisions were certainly made of an inch and a half in length, will one or even two incisions of fourteen inches in length arrest such an extent of disease? My experience teaches me that they will not, and hence it is, that I have stated, that in such desperate cases we must have recourse to such a number of small incisions, according to the extent of inflamed surface, as will arrest the disease. It appears to me more than probable that the fatal case of erysipelas treated after this manner, and detailed by Mr. Lawrence with so much candour, might not have terminated so, had the incision been confined to an inch or an inch and a half; for it was in this case, if I mistake not, that he made his longest incision; but even here the patient ought not to have been lost, for surely the dresser, or house-surgeon of the hospital, might have secured the bleeding vessels with a ligature: and therefore this case ought not to militate against the practice." 220.

All the world knows how wittily Mr. Lawrence repelled this sortie of his learned friend, proving that the incisions, "by instalment," did actually exceed *in length* the wholesale cuts which were represented as so dangerous in the hands of Mr. Lawrence. We have no doubt that each of the three modifications (for they are only modifications of the same practice) will be occasionally applicable, and not only applicable but preferable, one to the others, in particular cases. The judicious practitioner with not have much difficulty in making the selection. In this article we have concentrated a mass of information on a very important point of surgery, which, we hope, will prove useful to our readers, on both sides of the Atlantic.
